# Differential AMPK-mediated metabolic regulation observed in hibernation-style polymorphisms in Siberian chipmunks

**DOI:** 10.3389/fphys.2023.1220058

**Published:** 2023-08-16

**Authors:** Taito Kamata, Shintaro Yamada, Tsuneo Sekijima

**Affiliations:** ^1^ Graduate School of Science and Technology, Niigata University, Niigata, Japan; ^2^ Faculty of Agriculture, Niigata University, Niigata, Japan; ^3^ Institute of Biomedical Science, Kansai Medical University, Osaka, Japan

**Keywords:** Acc, AMPK, brain, chipmunk, circannual rhythm, hibernation, hypothalamus, Polymorphisms

## Abstract

Hibernation is a unique physiological phenomenon allowing extreme hypothermia in endothermic mammals. Hypometabolism and hypothermia tolerance in hibernating animals have been investigated with particular interest; recently, studies of cultured cells and manipulation of the nervous system have made it possible to reproduce physiological states related to hypothermia induction. However, much remains unknown about the periodic regulation of hibernation. In particular, the physiological mechanisms facilitating the switch from an active state to a hibernation period, including behavioral changes and the acquisition of hypothermia tolerance remain to be elucidated. AMPK is a protein known to play a central role not only in feeding behavior but also in metabolic regulation in response to starvation. Our previous research has revealed that chipmunks activate AMPK in the brain during hibernation. However, whether AMPK is activated during winter in non-hibernating animals is unknown. Previous comparative studies between hibernating and non-hibernating animals have often been conducted between different species, consequently it has been impossible to account for the effects of phylogenetic differences. Our long-term monitoring of siberian chipmunks, has revealed intraspecific variation between those individuals that hibernate annually and those that never become hypothermic. Apparent differences were found between hibernating and non-hibernating types with seasonal changes in lifespan and blood HP levels. By comparing seasonal changes in AMPK activity between these polymorphisms, we clarified the relationship between hibernation and AMPK regulation. In hibernating types, phosphorylation of p-AMPK and p-ACC was enhanced throughout the brain during hibernation, indicating that AMPK-mediated metabolic regulation is activated. In non-hibernating types, AMPK and ACC were not seasonally activated. In addition, AMPK activation in the hypothalamus had already begun during high Tb before hibernation. Changes in AMPK activity in the brain during hibernation may be driven by circannual rhythms, suggesting a hibernation-regulatory mechanism involving AMPK activation independent of Tb. The differences in brain AMPK regulation between hibernators and non-hibernators revealed in this study were based on a single species thus did not involve phylogenetic differences, thereby supporting the importance of brain temperature-independent AMPK activation in regulating seasonal metabolism in hibernating animals.

## Introduction

Hibernation is a unique physiological phenomenon allowing extreme hypothermia in certain endothermic animals. Hibernating individuals switch behaviors and physiological states beyond Tb between active and hibernation phases (Geiser, 2013). Hibernating animals maintain hypothermia for days to weeks (Geiser and Ruf, 1995). In daily torpor, which is often compared to hibernation, the Tb drop is less than 12 h (Geiser and Ruf, 1995). Furthermore, hibernation is generally more seasonal than daily torpor, with hibernation occurring from fall to winter in many hibernating species (Geiser, 2020). The essential factor distinguishing the two is that the circadian clock does not control hibernation, unlike daily torpor (Ruf and Geiser, 2015). Mammalian hibernators are often typed by feeding strategies and driving mechanisms of hibernation onset. Regarding the driving mechanism, obligate hibernators (e.g., ground squirrels Spermophilus, marmots Marmotini, and bears Ursus), which are triggered by an endogenous factor such as circannual rhythm, and facultative hibernators (e.g., hamsters Mesocricetus), which are triggered by changes in environmental conditions such as food and day length (Jansky et al., 1984; Kortner and Geiser, 2000; Schwartz and Andrews, 2013; Chayama et al., 2016). The chipmunks Tamias generally belongs to the obligate hibernators; however, eastern chipmunk T. striatus has a variable depth of torpor under food conditions (Munro et al., 2005), and siberian chipmunk T. sibiricus has a daily torpor type as a regional variation (Masaki, 2005) and non-hibernating individuals (Kondo et al., 2006). Regarding feeding strategy, chipmunks and hamsters are food-storing, whereas ground squirrels, marmots and bears are fat accumulating (Vander wall, 1990; Humphries et al., 2003). Besides the physiology and behavioral ecology of hibernation, for example, resistance to adverse factors including fungi, viruses and radiation have been well-studied (Torke and Twente, 1977; Sharapov, 1984; Schwartz et al., 2015; Puspitasari et al., 2021). Understanding the systems that regulate seasonal switching is very interesting and has long been the subject of research on hibernating animals (for example, ground squirrel: Yurisova and Polenov (1979); Ren et al. (2022), hamster; Bartness and Wade (1985); Garidou et al. (2003), bear; Fedrov et al. (2014), bat; Callard et al. (1983). Until the 1980s, the approach was mainly based on humoral factors and anatomy (Dawe and Spurrier, 1972; Oeltgen et al., 1978; Hudson and Wang, 1979). Since the 1990s, advancements in molecular biology techniques have allowed for the investigation of various genes that undergo changes in relation to the hypothermic state of hibernation in tissues such as the brain and liver (Takamatsu et al., 1993; Srere et al., 1995; O’Hara et al., 1999). From the 2000s onwards, omics analyses have made it possible to comprehensively profile the physiological states of hibernating animals, revealing the expression of genes and proteins involved in low-temperature tolerance and energy metabolism during hibernation (Williams et al., 2006; Shao et al., 2010; Schwartz et al., 2013; Xu et al., 2013). Recently, there has been increased interest in hibernation and daily torpor state, which is a period of reduced metabolism shorter than hibernation, in the fields of medicine and drug discovery. Approaches such as cell culture studies and manipulation of the nervous system have been utilized to analyze the intrinsic characteristics of hibernating cells and the mechanisms of low-temperature induction *in vivo* (Ou et al., 2018; Hrvatin et al., 2020).

Whether physiological and biomolecular changes during hibernation are the driving factors for hibernation or not, require careful discussion. Extreme hypothermia alters hundreds of gene expressions (Williams et al., 2006; Shao et al., 2010; Schwartz et al., 2013; Xu et al., 2013), and it is unlikely that all of the change factors are hibernation drivers. Therefore, simple comparisons of the changes in physiological states that occur with hibernation are likely to detect noise in elucidating the driving mechanisms of hibernation. Studies incorporating several perspectives can effectively test whether target factors are associated with hibernation drive. For example, Grabek et al. (2015) focused their validation on the phase immediately before the transition to hypothermia, in addition to the active and hibernating phases, to extract genetic changes independent of hypothermia. In addition, attempts have been made to consider the significance of their physiological mechanisms by comparing hibernating and non-hibernating animals (Caprette and Senturia, 1984; Matos-Cruz et al., 2017). However, for the former, there is the problem that the hibernation transition period is difficult to define, while for the latter, phylogenetic differences between different species are inevitably included. It is essential to consider physiological and phylogenetic differences when making comparisons to determine whether changes in specific factors are involved in driving hibernation.

We have previously focused on the metabolic regulator protein AMPK to elucidate the mechanism of metabolic regulation of hibernation during fluctuations in Tb. AMPK is an intracellular, trimeric protein kinase of α, β, and γ subunits (Kahn et al., 2005). This kinase is phosphorylated and activated when intracellular AMP/ATP increases. It maintains constant intracellular energy homeostasis by activating the catabolic pathway and inhibiting the anabolic pathway. Activated AMPK increases ATP synthesis by phosphorylating ACC, which is involved in fatty acid synthesis (Dyck et al., 1999). Furthermore, activated AMPK is known to suppress protein synthesis via phosphorylation of eEF2, which is involved in the translation process of protein synthesis (Browne et al., 2004). AMPK has also been reported to have cytoprotective effects via energy saving, such as ischemia tolerance and cancer suppression. More recently, it has become clear that changes in AMPK activity in the brain’s nerves also regulate systemic metabolisms, such as feeding and heat production, via the autonomic nervous system (López et al., 2016). We hypothesized that AMPK as a metabolic sensor may be involved in metabolic regulation of hypothermia tolerance in hibernation and in nervous system-mediated systemic metabolic inhibition, and we have conducted research on AMPK function during hibernation. We compared the expression levels of p-AMPK and p-ACC in different tissues of hibernating and active siberian chipmunks (Yamada et al., 2019). As a result, we have shown that p-AMPK and p-ACC are enhanced during hibernation, particularly in the brain, and may inhibit protein synthesis in brain cells via downstream factors such as eEF2. On the other hand, phosphorylation of AMPK was not observed in most of the peripheral tissues. In marmot, some studies have reported changes in p-AMPK in the hypothalamus during hibernation and while awakening from hibernation by administration of an AMPK agonist to the third ventricle (Florant et al., 2010). These results suggest that modulation of AMPK activity in the brain may be involved in the metabolic regulation of hibernation and hypothermia tolerance. However, it is not known whether these changes in AMPK activity are specific to hibernating animals and related to the driving of hibernation, or are linked to a further hypothermia-dependent response.

In this study, we approached the position of AMPK in the hibernation regulation of chipmunks from two perspectives. First, we examined whether the seasonal activation of AMPK was hibernation specific. A non-hibernating type of siberian chipmunk has been confirmed, which does not exhibit hibernation throughout its life span (Kondo et al., 2006). We focused on the polymorphisms of their hibernation style, expecting to be able to minimize phylogeny-dependent physiological differences. Criteria for classifying hibernation styles were established by long-term Tb monitoring. We tested whether hypothermia is induced by day length changes and stress, in order to prove that the non-hibernating type is not a reversible phenotype. We clarified whether the non-hibernating type activates AMPK seasonally, using the hibernating type as a control group. Second, we investigated whether changes in AMPK precede the induction of hibernation. To precisely define the hibernation transition phase, we prepared natural day length conditions and monitored blood HP levels known to induce hibernation in chipmunks (Kondo et al., 2006). By comparing the three phases of active, pre-hibernation, and hibernation, we clarified whether AMPK activation was a factor that was altered prior to the induction of hypothermia.

## Materials and methods

### Animals

Male siberian chipmunks less than 1 year of age were purchased from Arcland Sakamoto Co. The animals were housed individually at 23°C under 12 h-light/dark cycle conditions and provided a standard mouse diet and water *ad libitum*. The animals were maintained under these conditions for at least 6 months to acclimate to laboratory conditions. After checking their general health conditions, they were used in the experiments described below.

All experiments involving the animals complied with protocols that were reviewed by the Institutional Animal Care and Use Committee and approved by the President of Niigata University (Permit Number: Niigata Univ. Res. 13–2, 258–1, 399–1, 530, SA00106, SA00107, SA00228, and SA00738). All protocols performed followed the Guiding Principles for Care and Use of Laboratory Animals (NIH, United States).

### Determination of hibernation state by measuring surface Tb

Temperature logger measurements are common in hibernation studies; however, the effects of invasive procedures on the maintenance of a non-hibernating state in chipmunks remains unknown. Therefore, we investigated the relationship between surface and core Tb to establish a non-invasive method of determining hibernation state.

Paraffin-coated (Eto et al., 2018) temperature loggers (Thermochron G, KN Laboratories) were implanted in the abdominal cavity of anaesthetized animals. Five animals were kept at 23°C for 2 weeks after implantation, then transferred to conditions of constant 5°C and darkness and monitored for 30 days. We measured core Tb every 30 min, and measured surface Tb (using an infrared irradiation thermometer; IT-540N, HORIBA) 21 times per animal throughout the study period, matching the timing of core Tb measurements.

### Establishment of criteria for classifying hibernation style

To create typological criteria for hibernation styles (hibernating and non-hibernating types), we kept 324 animals under conditions of constant 5°C and darkness. We measured the surface Tb once daily (17:00–20:00). We placed wood chips on the body surface of hibernating animals to confirm whether they were still hibernating the next day. Food and water were provided *ad libitum*. Through lifelong monitoring, the number of days of cold exposure required to induce hibernation was determined.

### Hibernation induction under semi-natural conditions

To support that hibernating and non-hibernating types are not differences in animal decision-making but phenotypic irreversibility, we considered that validation under conditions other than 5°C and constant darkness be necessary. Therefore, we conducted a hibernation induction experiment by semi-natural conditions. Furthermore, this experiment will allow us to make seasonal comparisons of Tb for chipmunks whose life cycles are free-running under constant conditions such as 5°C and constant darkness (Kondo et al., 2006).

Initially, 11 animals were kept under 5°C and darkness for 190 days and Tb monitoring was performed to classify hibernating (n = 5) and non-hibernating types (n = 6). We moved the animals to semi-natural condition in April. The environment in this room is controlled by natural light and outside temperature. Day length and outside temperature changes during the experiment are shown in Supporting Information S1. In Niigata Prefecture, Japan, where the experiments were conducted, day length was approximately 10–15 h, and the range of temperature was −1.7°C–30.8°C (JMA, 2023; NAOJ, 2023) during the experiment. Room temperature was controlled not to exceed 25°C.

We set up three treatments: hibernating type (CT, n = 5) and non-hibernating type (NH, n = 3) with food and water provided *ad libitum*, and non-hibernating type with bi-daily water deprivation (NHW, n = 3). We conducted water deprivation from 21-September to 20-March under short-day conditions (12 h or less). Water deprivation has long been considered one of the external factors involved in the occurrence or non-occurrence of hibernation onset (Ibuka and Fukumura, 1997). Core Tb was measured with implanted loggers from September to May.

### Measurement of blood HP concentration

For comparison of HP regulation (as described by Kondo et al., 2006), we kept hibernating (n = 11) and non-hibernating types (n = 5) under 5°C and darkness for 24 months. We collected blood samples from the lateral tarsal vein of experimental animals once a month. Samples of approximately 400 µl of blood were collected, and plasma was separated using a centrifuge. To prevent clotting during blood collection, 10 µl of heparin was mixed with the blood. The separated plasma was stored at −80°C until the concentration was measured. The Western blot method was used to measure the concentration of HPs (HP-20, HP-25, and HP-27) in the blood.

### Tissue sampling

As the non-hibernating type does not exhibit hypothermia, it is difficult to identify the phases corresponding to the transition from the active season to the hibernating season in this type. Tissue sampling was therefore conducted under semi-natural conditions. We sampled hibernating types in three phases: active, pre-hibernation, and hibernation. The timing of sample-taking was guided by Tb and blood HP levels (Supporting Information S2). HP is a factor related to hibernation regulation in chipmunks and is known to decrease blood levels prior to hibernation (Kondo and Kondo, 1992). For the non-hibernating type, only the month was used as a guide, as neither Tb nor blood HP changes. Therefore, we sampled active hibernating and non-hibernating types in summer, hibernating type during the pre-hibernation state in autumn, and both hibernating and non-hibernating types in winter.

We anaesthetized the animals using CO_2_ then euthanized them by decapitation. After craniotomy, the brain was divided on ice into the cerebral cortex, hippocampus, cerebellum, diencephalon, and hypothalamus regions. The heart, liver, kidneys, and skeletal muscle were sampled for peripheral tissues after opening the abdomen. Each tissue sample was frozen on dry ice and stored at −80°C.

## Materials

Anti-phospho-AMPKα (Thr172), anti-phospho-ACC (Ser79), were purchased from Cell Signaling Technology. Validation of their antibody specificity to chipmunk proteins has been confirmed in a previous study (Yamada et al., 2019). The amino acid sequence of AMPKα1, the phosphorylation site of AMPK, the target molecule in this study, has also been shown to be well conserved among species (Yamada et al., 2019). We used the same antibodies that were used by Kondo et al. (2006), having received them from Dr. Kondo. HP is a protein that forms a complex of three types: −20, −25, and −27. As previous studies (Kondo and Kondo, 1992; Takamatsu et al., 1993) have shown that the blood cycles of any HP are synchronized, in this study we used the HP-20 antibody to measure the hibernation cycle of chipmunks.

### Western blotting

Tissue extracts and Western blotting were performed as described previously by Yamada et al. (2019). Frozen tissue samples were weighed and homogenized in ten volumes of lysis buffer (62.5 mM Tris-HCl (pH 6.8), 2% SDS, Complete protease inhibitor cocktail (Roche Applied Science Ltd.), and PhosStop (Roche Applied Science). After centrifugation (15,000 rpm × 60 min), supernatants were collected. The protein concentration of each sample was determined by Lowry (BIO-RAD). For plasma samples, a 100-fold volume of lysis buffer (62.5 mM Tris-HCl (pH 6.8), 2% SDS, Complete protease inhibitor cocktail (Roche Applied Science) was used.

Equal amounts of protein (30–50 μg per lane for indicated molecules) were subjected to sodium dodecyl sulfate-polyacrylamide gel electrophoresis (7.5%–15% acrylamide) and transferred to polyvinylidene fluoride membranes. Plasma samples were loaded to 10 nl per lane and electrophoresed in the same manner as tissue samples. Membranes were cropped around the appropriate molecular size to save the amount of antibodies. The membranes were blocked in TNT (150 mM NaCl, 10 nM Tris-HCl (pH 7.4), and 0.05% tween-20) containing 10% BSA and then incubated with the indicated primary antibody overnight. After washing with TNT, blotted membranes were incubated with horseradish-peroxidase (HRP)-conjugated anti-rabbit IgG (1:10000 dilution; Dako Cytomation) or HRP-conjugated anti-mouse IgG (1:10000 dilution; Jackson Immune Research) for 1 h. After washing, peroxidase activity was detected by chemiluminescence reagents (Western Lightning, PerkinElmer Life Science) and visualized on X-ray film (Fujifilm Medical). The quantity of protein expressed was quantified by ImageJ software and standardized by β-actin.

### Statistical analysis

We used R ver. 4.2.1 for all statistical analyses (R Core Team, 2023). Log-Rank tests were used to compare survival rates between hibernating and non-hibernating types, and T-tests were used for all two-group comparisons. For all multi-group comparisons we used analysis of variance (ANOVA) and Tukey HSD test. To demonstrate circannual rhythmicity, we Fourier transformed blood HP levels for 24 months and calculated frequencies (month) and amplitudes. We showed the mean ± SE in results.

## Results

### Relationship between core and surface Tb

Under conditions of 5°C and darkness, surface Tb during hibernation was in the range 3.7°C–7.9°C, and core Tb was in the range 4.5°C–8.0°C, whereas during arousal surface Tb was in the range 14.9°C–23.4°C and core Tb was in the range 36.0°C–38.5°C (Supporting Information S3A). Hibernating and arousal states could be clearly distinguished because Tb ranges did not overlap. Therefore, we defined hibernation state as surface Tb below 10°C and arousal state as above 11°C. This criterion allowed non-invasive monitoring of hibernating types that hibernate periodically and non-hibernating types that never hibernate (Supporting Informations S3B, C).

### Criteria for classifying hibernation styles

**FIGURE 1:**
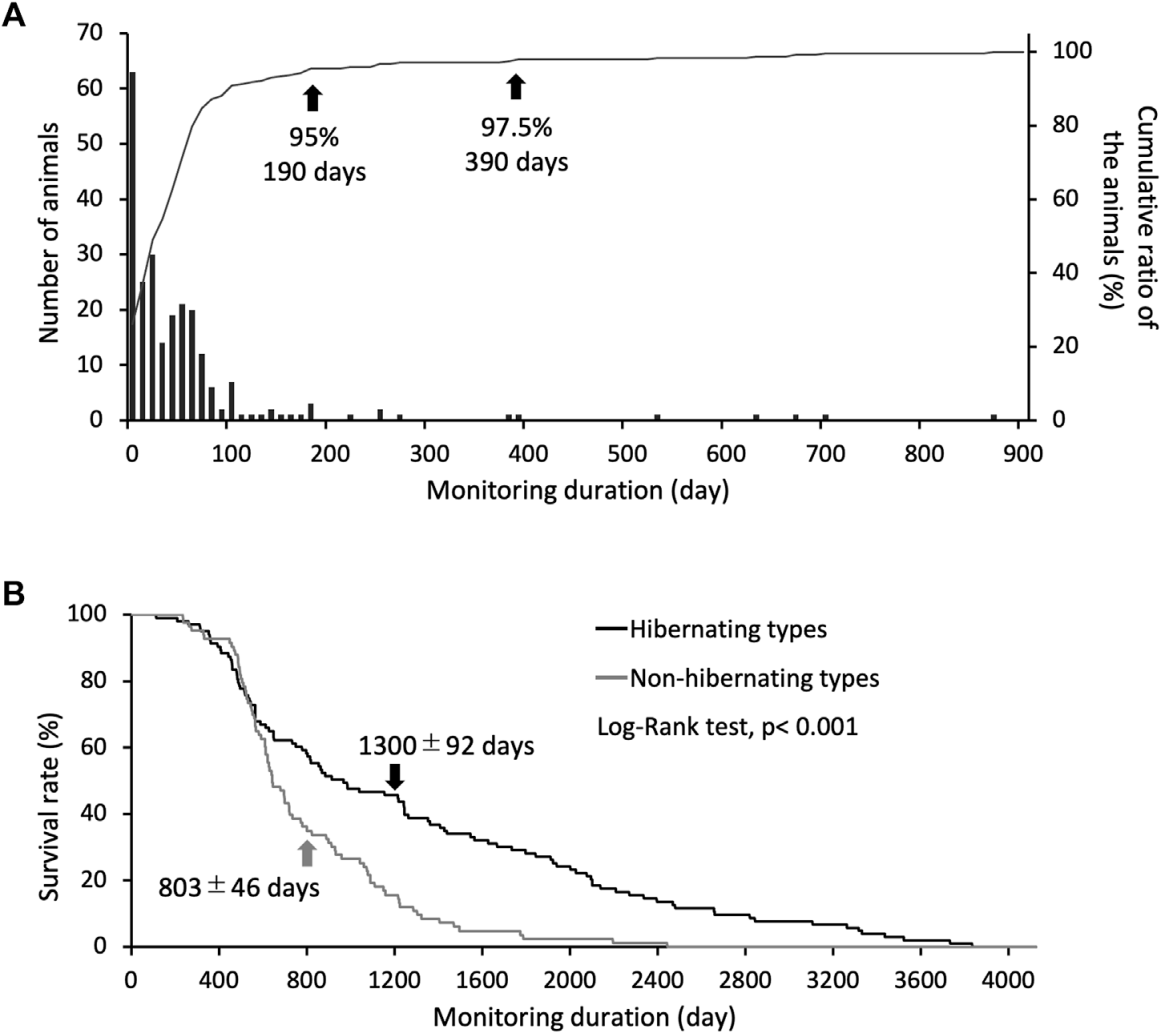
Criteria for identifying hibernation styles through long-term hibernation monitoring. **(A)** bars show the number of animals for each day required to reach hibernation induction, and the line shows the cumulative ratio of the animals hibernated (only use hibernating types, n = 241). Arrows indicate the number of days for the classification accuracy of hibernating and non-hibernating types. **(B)** Shows the difference in survival rates between individual hibernating (n = 103) and non-hibernating chipmunks (n = 83), with a significant difference between them (Log-Rank test, *p* < 0.001). Arrows show mean ± SE.

Induction of hypothermia was identified in 241 of 324 study animals, of which 95% became hypothermic and hibernated after 190 days, and 97.5% became hypothermic and hibernated after 390 days (Figure 1A). Eighty-three non-hibernating animals were identified. The life span of the hibernating type (1,300 ± 92 days) was approximately 1.7 times that of the non-hibernating type (803 ± 46 days), indicating a significant difference in survival rates between the two types (Figure 1B). Individuals used for life-span analysis were kept at 5°C throughout their lives.

### Variation of HP regulation between hibernation styles

**FIGURE 2:**
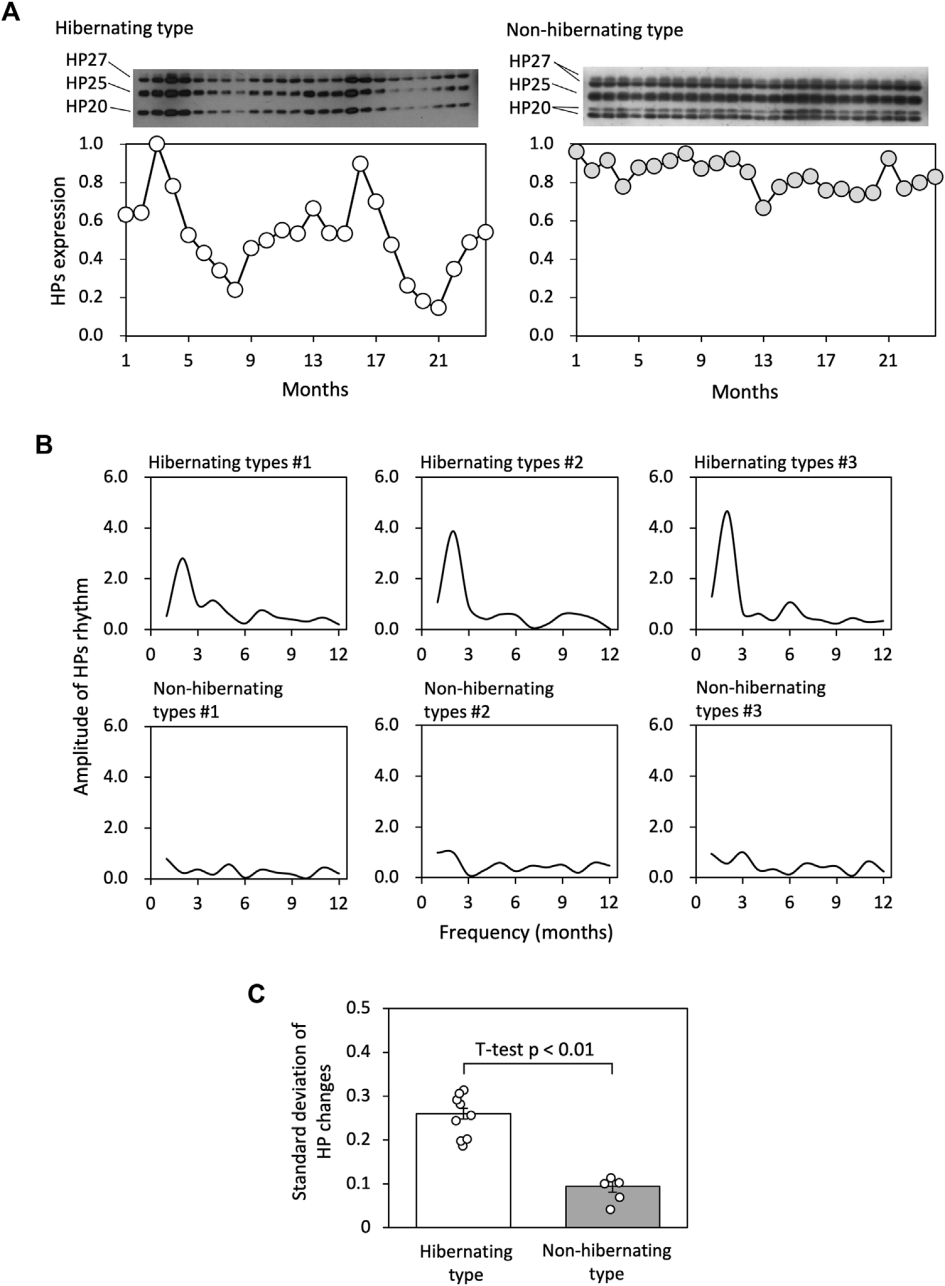
Comparison of HP regulation between hibernating and non-hibernating types. **(A)** Shows typical HPs (HP-20, HP-25, and HP-27) fluctuation patterns of hibernating and non-hibernating types under conditions of 5°C and constant darkness. **(B)** Shows the amplitude values for frequency (months) of the HPs rhythm for 24 months by Fourier transformation in hibernating and non-hibernating types. **(C)** Shows the standard deviation of HPs level for hibernating (n = 11) and non-hibernating types (n = 5).

HPs fluctuation patterns of hibernating and non-hibernating types under conditions of 5°C and darkness were studied for 2 years (see Figure 2A). Fourier transformation of the 24-month HPs levels showed that the most substantial amplitudes for the hibernating type were at frequencies of approximately 12 months. In contrast, the amplitudes were weaker for the non-hibernating type (Figure 2B). we focused on the standard deviations of HPs changes and found significant differences between hibernating and non-hibernating types (Figure 2C).

### Non-hibernating type as an irreversible phenotype

**FIGURE 3:**
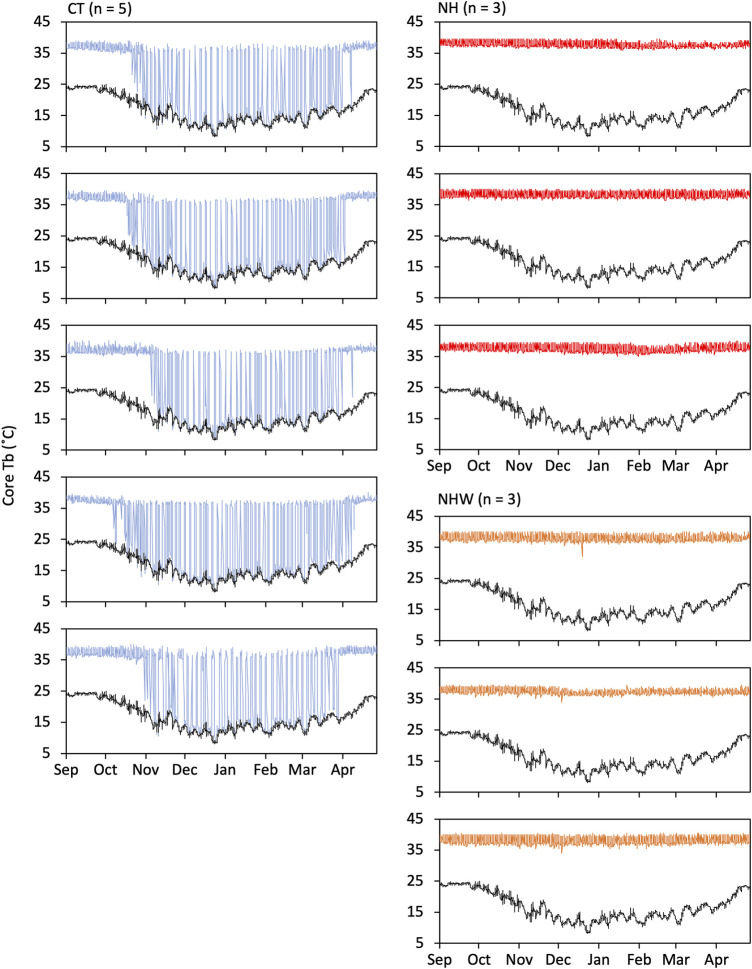
Hibernation induction under semi-natural conditions. Hibernating and non-hibernating types classified by monitoring under conditions of 5°C and darkness after being moved to semi-natural conditions. We investigated whether non-hibernating types hibernate under natural environmental conditions (temperature and day length changes) and bi-daily water deprivation (from 23 September to 21 March). Tb changes of all animals used in the experiment are shown in figure. Specify colored line indicates Tb and black line indicate the ambient temperature. CT (hibernation type, blue), NH (non-hibernating type, red), and NHW (non-hibernating type with water deprivation, orange).

The hibernating type showed seasonal hibernation induction, whereas the non-hibernating type did not become hypothermic, even under water stress (Figure 3). Mean value ± SE of minimum Tb measurements throughout the experimental period were 8.6°C ± 0.1°C for CT, 35.8°C ± 0.4°C for NH, and 33.3°C ± 0.7°C for NHW. The mean value of parameters on hibernation (only CT) were as follows: hibernation began on 2 November ± 5.1 days and ended on 15 April ± 2.5 days; 54.6 ± 4.4 times of bout were recorded with a maximum hibernation bout of 94.4 ± 2.7 h; mean hibernation duration was 164 ± 6.2 days.

**FIGURE 4:**
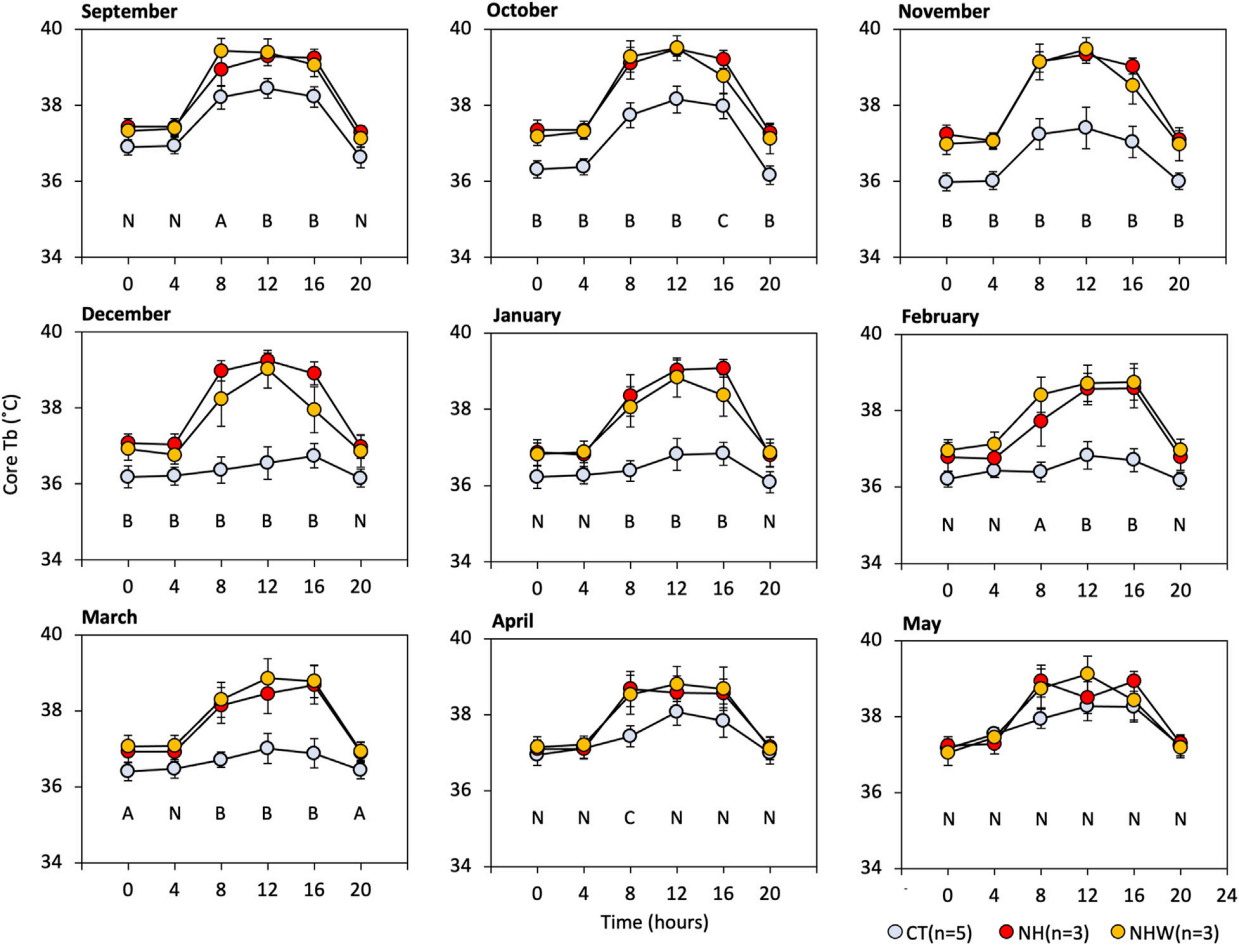
Monthly arousal Tb under semi-natural conditions. We extracted core Tb every 4 hours while subjects were awake (≥35°C). Specify colored circle indicates arousal Tb. CT (hibernation type, blue), NH (non-hibernating type, red), and NHW (non-hibernating type with water deprivation, orange). Letters indicate multiple comparisons with statistical significance (N, no significance in all combinations; A, CT-NHW; B, CT-NH, and−NHW; C, CT-NH), *p* < 0.05 Tukey HSD after ANOVA). Error bars indicate SE.

We focused on Tb during the arousal state, so as to allow for comparisons between hibernating and non-hibernating types (Figure 4). Significant differences between treatments were found between September (before hibernation) and April (after hibernation ended). No significant differences were found in May, the active season. The most significant divergence in arousal temperature between hibernating and non-hibernating types (a difference of approximately 2.5°C) was during the mid-hibernation period, in December. All groups showed diurnal synchronized changes in Tb, which increased during the day and decreased at night, with the differences between groups being greater during the day (08:00–16:00) than at night (16:00–08:00).

### Comparison of AMPK activation between hibernation style polymorphisms

**FIGURE 5:**
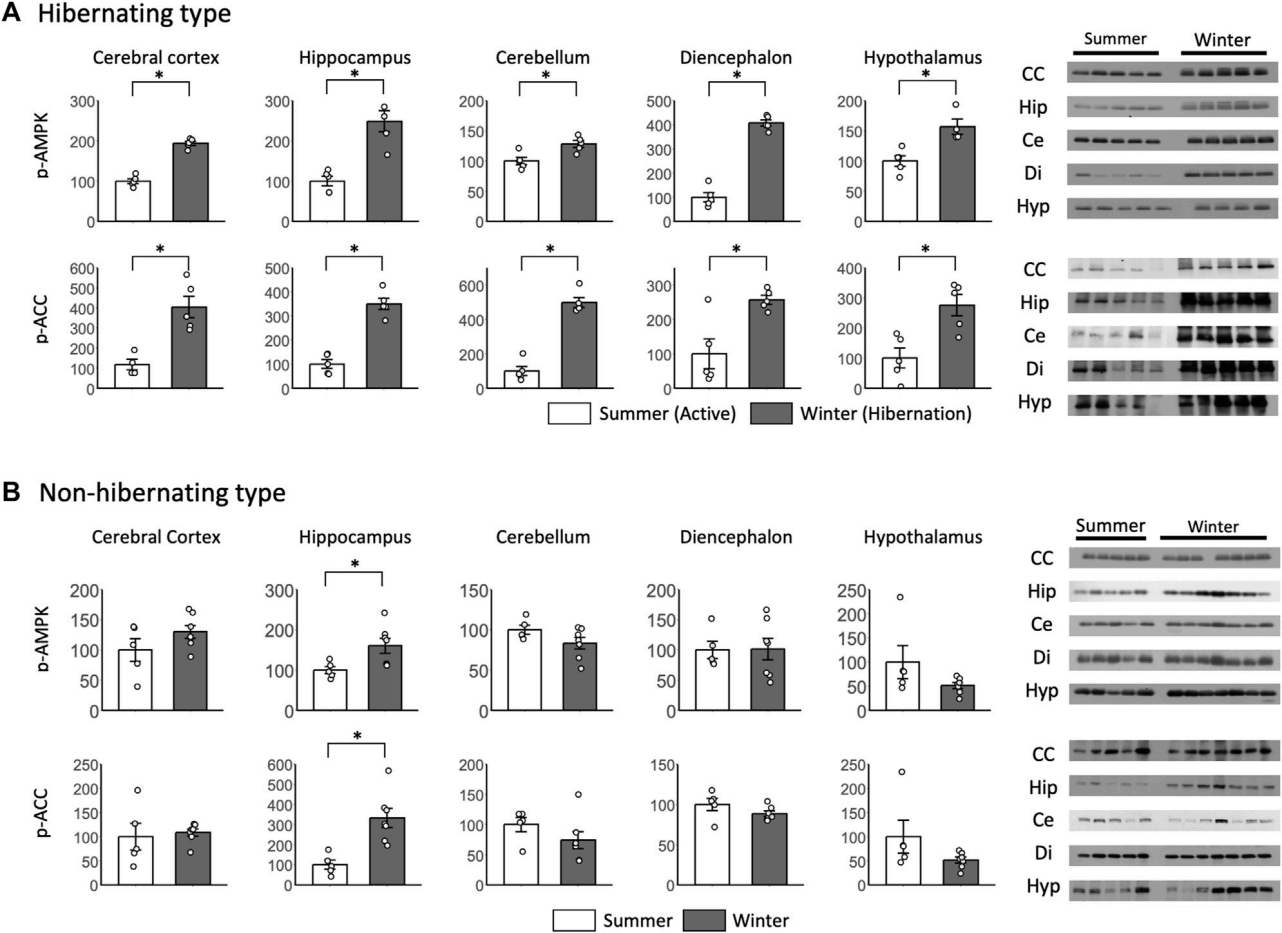
Comparison of phosphorylated AMPK and ACC expression in the brain between hibernating and non-hibernating types. Expression levels of phosphorylated AMPK and ACC (p-AMPK and p-ACC) in brain tissues (cerebral cortex, hippocampus, cerebellum, diencephalon, and hypothalamus) of hibernating **(A)** and non-hibernating types **(B)**. Error bar means SE. Hibernating type: active n = 5 (hypothalamus only n = 4), Hibernating n = 5, non-hibernating type summer n = 5, winter n = 7. CC: Cerebral Cortex; Hip: Hippocampus; Ce: Cerebellum; Di: Diencephalon; Hyp: Hypothalamus. *p < 0.05 student’ t-test. Internal control see Supporting Information S4A.

p-AMPK and p-ACC were enhanced in all brain regions during hibernation, and significant differences were found between summer and winter in the hibernating type (Figure 5A). For p-AMPK and p-ACC of the non-hibernating type, significant differences were found between summer and winter only in the hippocampus, which was enhanced in winter (Figure 5B).

**FIGURE 6:**
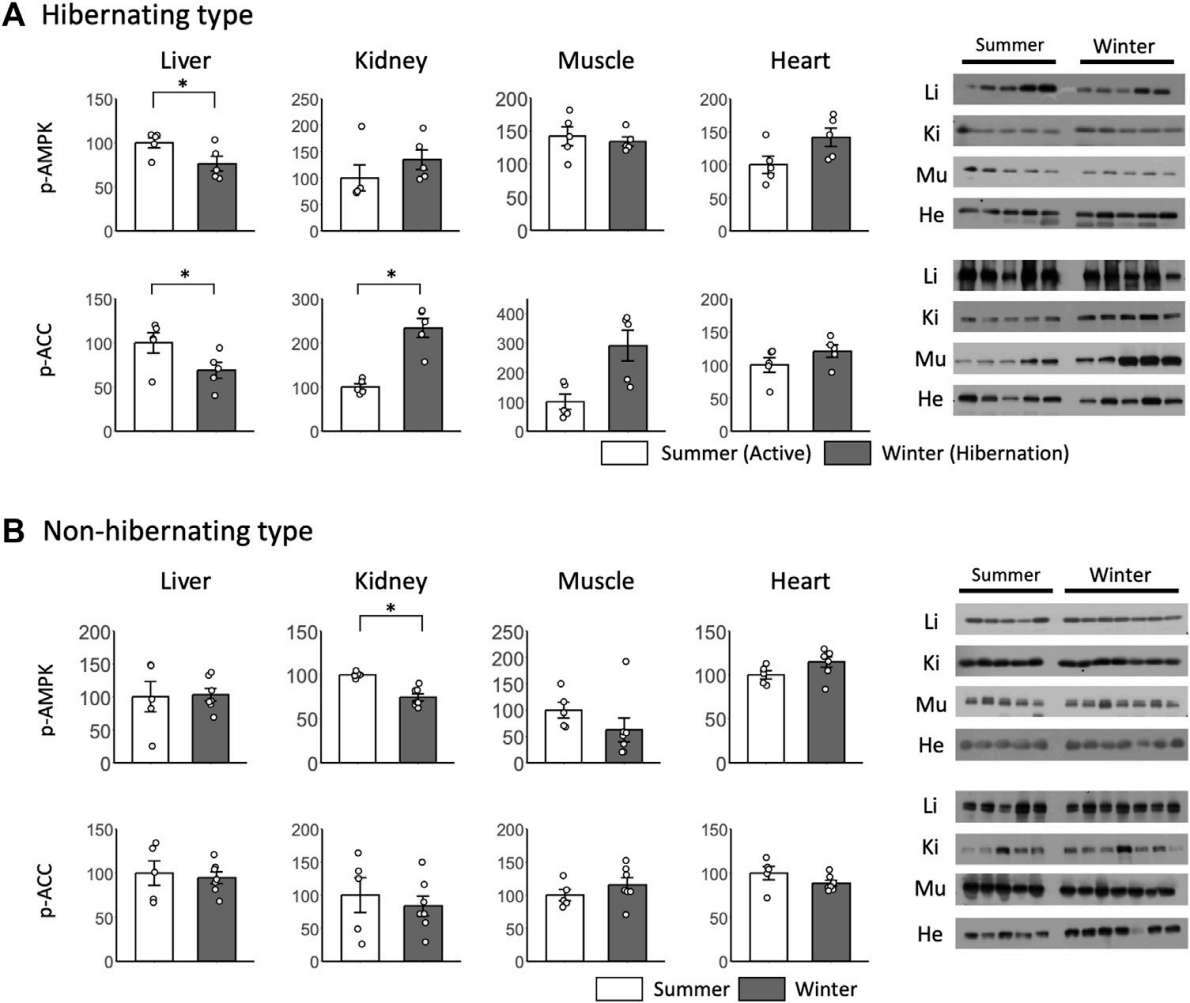
Comparison of phosphorylated AMPK and ACC expression in peripheral tissues between hibernating and non-hibernating types. Expression levels of phosphorylated AMPK and ACC (p-AMPK and p-ACC) in peripheral tissues (liver, kidney, muscle, heart) of hibernating **(A)** and non-hibernating types **(B)**. Error bar means SE. Hibernating type: summer n = 5, winter n = 5 (hypothalamus only n = 4), non-hibernating type summer n = 5, winter n = 7. Liv, Liver; Kid, Kidney; Mus, Muscle; Hea, Heart. *p < 0.05 student’ t-test. Internal control see Supporting Information S4A.

For peripheral tissues during hibernation, no significant differences in p-AMPK were found between summer and winter for almost all tissues (Figure 6A). p-AMPK expression was decreased only in the liver. For p-ACC, significant differences between summer and winter were found only in the liver and kidney, with a decrease in the liver and an increase in the kidney during winter (Figure 6A). For the non-hibernating type, a significant difference between winter and summer was found only in p-AMPK in the kidneys (Figure 6B).

### AMPK activation in the pre-hibernation period

**FIGURE 7:**
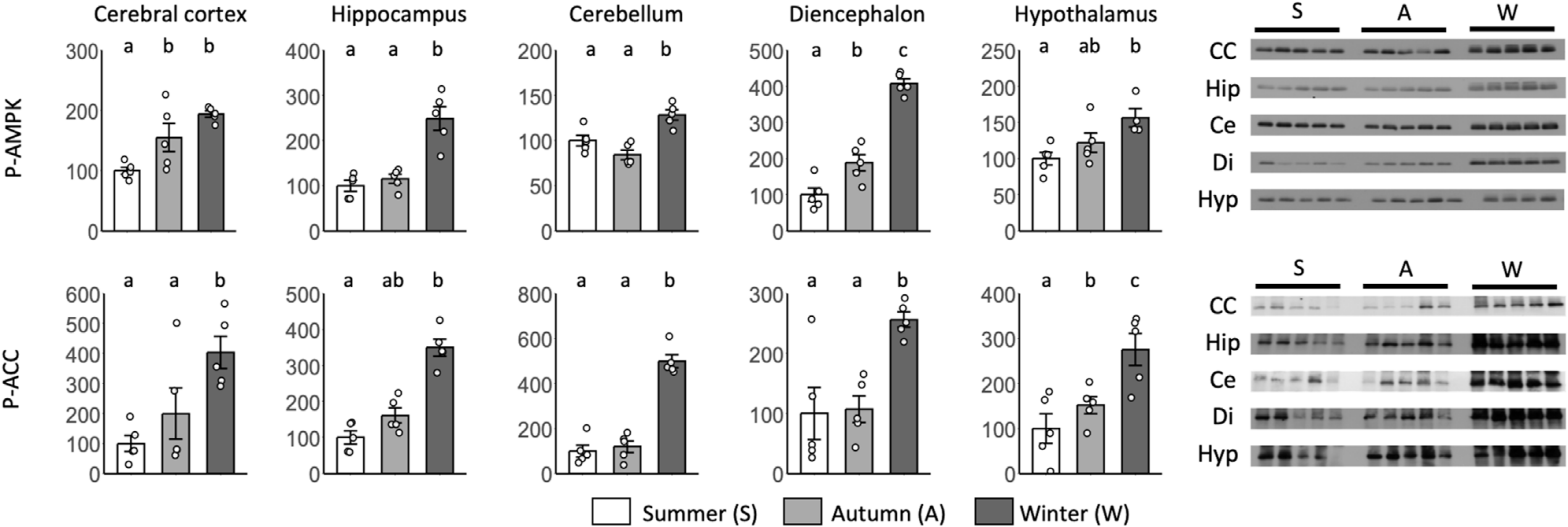
Seasonal AMPK and ACC expression in the brain during the hibernation transition. Expression levels of phosphorylated AMPK and ACC (p-AMPK and p-ACC) in brain tissues (cerebral cortex, hippocampus, cerebellum, diencephalon, and hypothalamus) during summer, autumn, and winter. Error bar means SE. Hibernation type: summer n = 5 (hypothalamus only n = 4), autumn n = 5, winter n = 5. Letters indicate significant differences, *p* < 0.05 Tukey HSD after ANOVA. CC, Cerebral Cortex; Hip, Hippocampus; Ce, Cerebellum; Di, Diencephalon; Hyp, Hypothalamus. Internal control see Supporting Information S4B.

Significant differences between summer and autumn were found for p-AMPK in the cerebral cortex and diencephalon and for p-ACC in the hypothalamus (Figure 7). Significant differences between autumn and winter were found for p-AMPK in the diencephalon and p-ACC in the hypothalamus. Although no significant differences were found, p-AMPK levels in the hypothalamus and p-ACC levels in the cortex and hippocampus tended to be greater in autumn than in summer.

## Discussion

### Hibernation style polymorphisms

Several studies of intraspecific variation in hibernation have been conducted on Sciuridae. Among ground squirrel species, hibernation periods have been reported to vary regionally (Zervanos et al., 2010; Sheriff et al., 2011; Grabek et al., 2019). Variation among individuals within the same population has been reported in the eastern chipmunk, the Tb of the hibernating state and energy reserves determine the torpor frequency and depth (Munro et al., 2005; Levesque and Tattersall, 2010). Siberian chipmunks from the Korean (T. s. barberi) and Japanese populations (T. *s. lineatus*) have different hibernation patterns under the same keeping conditions, with shorter bouts in the former and longer ones in the latter (Masaki, 2005). The clear emergence of hibernating and non-hibernating types, even in the same keeping condition as in our study, is extremely rare in hibernation studies to date. Whether non-hibernating types behave in the field has not been confirmed, however, Turbill and Prior (2016) estimate that survival rates for non-hibernating rodents are higher and hibernating rodents lower in warmer environments. Siberian chipmunk, which has a wide range in Eurasia (Lissovsky et al., 2017), may be regionally adapted by having a polymorphism of various hibernation styles. Investigating the distribution, abundance ratios, and phylogeny of the polymorphisms in the field are future works.

We found the lack of a blood HP rhythm in non-hibernating types to be evident, as did Kondo et al. (2006) in a previous study, and it seems likely that there are mutations in the integrative regulatory systems involved in HP transport to the brain. Pseudogenization of HP in the family Sciuridae results in a lack of hibernation ability (Takamatsu et al., 1993; Kojima et al., 2001; Ono et al., 2003; Tsukamoto et al., 2007), indicating that HP regulation is involved in the mechanisms that determine hibernation style. The presence or absence of HP brain transport in the non-hibernating type is a target for future research.

In syrian golden hamsters *Mesocricetus auratus* and arctic ground squirrels Urocitellus parryii, it has been reported that decreased arousal Tb is related to the hibernation cycle (Olson et al., 2013; Chayama et al., 2016). The relationship between hypometabolism on arousal and extreme hypothermia, such as in hibernation and daily torpor, has been tested by administering N6-Cyclohexyladenosine (CHA) to starved rats and ground squirrels (Jinka et al., 2010; Jinka et al., 2011; Olson et al., 2013). Sensitivity to CHA is increased at lower Tb levels, which has been shown to result in more significant temperature reductions than at high Tb levels. Mechanisms that allow hypothermia may be associated with reduced basal Tb. A lack of such a function may be inferred in the non-hibernating type.

Little is known about the genetic regulatory mechanisms involved in intraspecific variation in hibernation style. Grabek et al. (2019) recently showed that genetic factors are responsible for intraspecific variation in the hibernation period of thirteen-lined ground squirrel Ictidomys tridecemlineatus. However, there have been no reports on the genetic factors determining hibernation or non-hibernation within the same species. To address this subject, we have established chipmunk breeding methods under laboratory conditions and have successfully carried out planned crosses (Kamata et al., 2020). In the future we aim to use breeding and positional cloning to elucidate the mode and rate of inheritance of hibernation-style polymorphisms, and search for the dominant genes involved in the hibernation drive.

### Is AMPK activation related to a driving factor for hibernation?

AMPK activation in hibernating animals has been reported to be enhanced in the ground squirrel’s adipocytes (Horman et al., 2005) and in the chipmunk brain’s cerebral cortex and hypothalamus (Yamada et al., 2019). AMPK activation in the liver was inconsistent, with some enhanced and some suppressed (Yamada et al., 2019). Unlike Yamada et al. (2019), the present study found enhancement in the whole brain (cerebral cortex, hippocampus, cerebellum, diencephalon, and hypothalamus), and we consider that the inconsistency in responses is likely related to rearing conditions. In Yamada et al. (2019), study animals were maintained at 5°C and in constant darkness, while in this study, they were maintained at ambient temperature and natural day length. The hibernation cycle of chipmunks is free-running under constant-dark conditions (Kondo et al., 2006) and is adjusted by natural day length (Figure 3). The metabolic regulator Akt, which shares a downstream factor with AMPK, is known to change its phosphorylation state depending on the phase of hibernation regulation (Cai et al., 2004). The more pronounced phosphorylation of AMPK in this study may be because AMPK phosphorylation by the hibernation cycle was detected more accurately than by Yamada et al. (2019).

The seasonal activation of AMPK in the brain occurred according to circannual rhythms and disappeared with the lack of the rhythms, which strongly supports a relationship between AMPK and hibernation. AMPK activation in hibernation is not independently influenced by day length and would be regulated by endogenous mechanisms. As for the peripheral tissues, AMPK activation may not be essential for metabolic regulation in hibernation, as pointed out in a previous study (Horman et al., 2005). However, previous studies (Horman et al., 2005; Yamada et al., 2019) as well as the current study consistently observed the suppression of AMPK phosphorylation in the liver during hibernation. This suggests that AMPK may be suppressed by regulatory functions to strongly promote fatty acid synthesis as an energy storage source, as Horman et al. (2005). In the non-hibernating type, phosphorylation of AMPK and ACC was found during hibernation only in the hippocampus, despite the absence of AMPK phosphorylation in most tissues. It is well known that the hippocampus is the brain region primarily important for memory, and AMPK has been reported to have memory-related functions in the hippocampus (Zhou et al., 2023). Several studies on memory during hibernation have been reported. In particular, male chipmunks have been reported to memorize a female’s den before hibernation for the early spring mating season and to be able to maintain this memory during hibernation (Kawamichi and Kawamichi, 1993). Thus, the phosphorylation of AMPK and ACC in the hippocampus of non-hibernating chipmunks as well as hibernating chipmunks may suggest that signaling systems related to neural energy utilization and seasonal regulation related to neural plasticity in relation to memory are driven by a different system than hibernation.

Since physiological changes that precede hibernation begin before hypothermia (Kondo, 1987; Grabek et al., 2015), the pre-hibernation state is considered an essential phase for elucidating the driving mechanisms. *In vitro* assays revealed that AMPK is activated at low temperatures (Knight et al., 2015); however, our study found a non-temperature dependence of seasonal AMPK activation in the hypothalamus and mesencephalon. AMPK functions in the brain include acting on downstream factors such as eEF2, mTOR, and ACC to inhibit protein and lipid synthesis and reduce wasteful energy consumption (Kahn et al., 2005). Furthermore, these functions are known to have protective effects on neurons, including ischemia tolerance, by suppressing reactive oxygen species and energy starvation and promoting autophagy. Since hypothermia is known to cause leakage of neurotransmitters and damage to mitochondria and cytoskeleton by reactive oxygen species (Schwartz et al., 2013; Ou et al., 2018), activation of AMPK during the hypothermic phase of hibernation may have a protective effect on neurons from low temperature and energy starvation. On the other hand, it has been noted that excessive AMPK activation can also cause excessive activation of autophagy and destroy neurons (Li et al., 2011), which will need to be verified in the future. AMPK has recently been shown to be involved in feeding and body thermoregulation, in addition to its function in neurons alone, by being activated in the diencephalon and hypothalamus (López et al., 2016). Activation of AMPK in the hypothalamus produces heat in brown adipose tissue and other tissues via sympathetic inhibition, and activation of AMPK in the Arcuate Nucleus enhances feeding (López et al., 2016). AMPK agonists induced feeding and inhibited hibernation in marmots, which fast during hibernation (Florant et al., 2010). In chipmunks, in contrast, known to feed during hibernation, activation of AMPK in the hypothalamus may be essential for maintaining feeding. AMPK is activated in the hypothalamus and mesencephalon before and during hibernation when basal body temperature decreases (Figure 4). Suppression of thermogenesis in brown adipose tissue via activation of AMPK (López et al., 2016) is associated with hibernation. It may contribute to increased brown adipose tissue before hibernation (MacCannell et al., 2017). Although it is not clear in this study whether AMPK affects the hypothermia induction of hibernation, in the future, intracerebroventricular administration experiments, as well as detailed behavioral and temperature monitoring, will clarify the relationship between AMPK activation and the function of hibernation.

AMPK activation during starvation is a passive response dependent on external factors (Kajita et al., 2008; Healy et al., 2011). In contrast, AMPK activation associated with the hibernation cycle is an active regulatory system, that is, adjusted by external factors (day length and ambient temperature). The variation of AMPK seasonality and the lack of HP rhythm may be vital to revealing the driving mechanisms of hibernation. Adiponectin, which is homologous to HP (Fujita et al., 2013), is one of the top factors of AMPK (Scherer et al., 1995; Kubota et al., 2007). We may gain a deeper understanding of the role of AMPK in driving hibernation by looking at the response of AMPK and its downstream factors after intracerebroventricular administration of HP.

## Conclusion

In this study, we developed classification criteria for hibernating and non-hibernating types of Siberian chipmunks and identified variations in the seasonality and HP rhythm of thermoregulation. Through comparisons among the revealed hibernation polymorphisms, it was shown that AMPK, a sensor of metabolic regulation, is likely to be involved in hibernation metabolism, especially in the brain. Furthermore, validation in the pre-hibernation period revealed that AMPK activation in the diencephalon and hypothalamus occurs prior to hibernation. Considering the seasonal trends in body temperature of chipmunks, the HP rhythm, and the function of AMPK, it is suggested through this study that AMPK is likely involved in systemic metabolic regulation important for hibernation via the autonomic nervous system and in hypothermia tolerance during hibernation.

## Data Availability

The original contributions presented in the study are included in the article/Supplementary Material, further inquiries can be directed to the corresponding author.

## Abbreviations

AMPK, AMP-activated protein kinase; ACC, Acetyl-CoA carboxylase; eEF2, eukaryotic elongation factor 2HP hibernation-specific protein; mTOR, mammalian target of rapamycin; p-AMPK, phosphorylated AMPK; p-ACC, phosphorylated ACC; Tb, body temperature.
