# Supplementary figures and images for: Differential AMPK-mediated metabolic regulation observed in hibernation-style polymorphisms in Siberian chipmunks
Source: Front Physiol. 2023 Aug 16;14:1220058. doi: 10.3389/fphys.2023.1220058 (PMC10468594; doi:10.3389/fphys.2023.1220058)

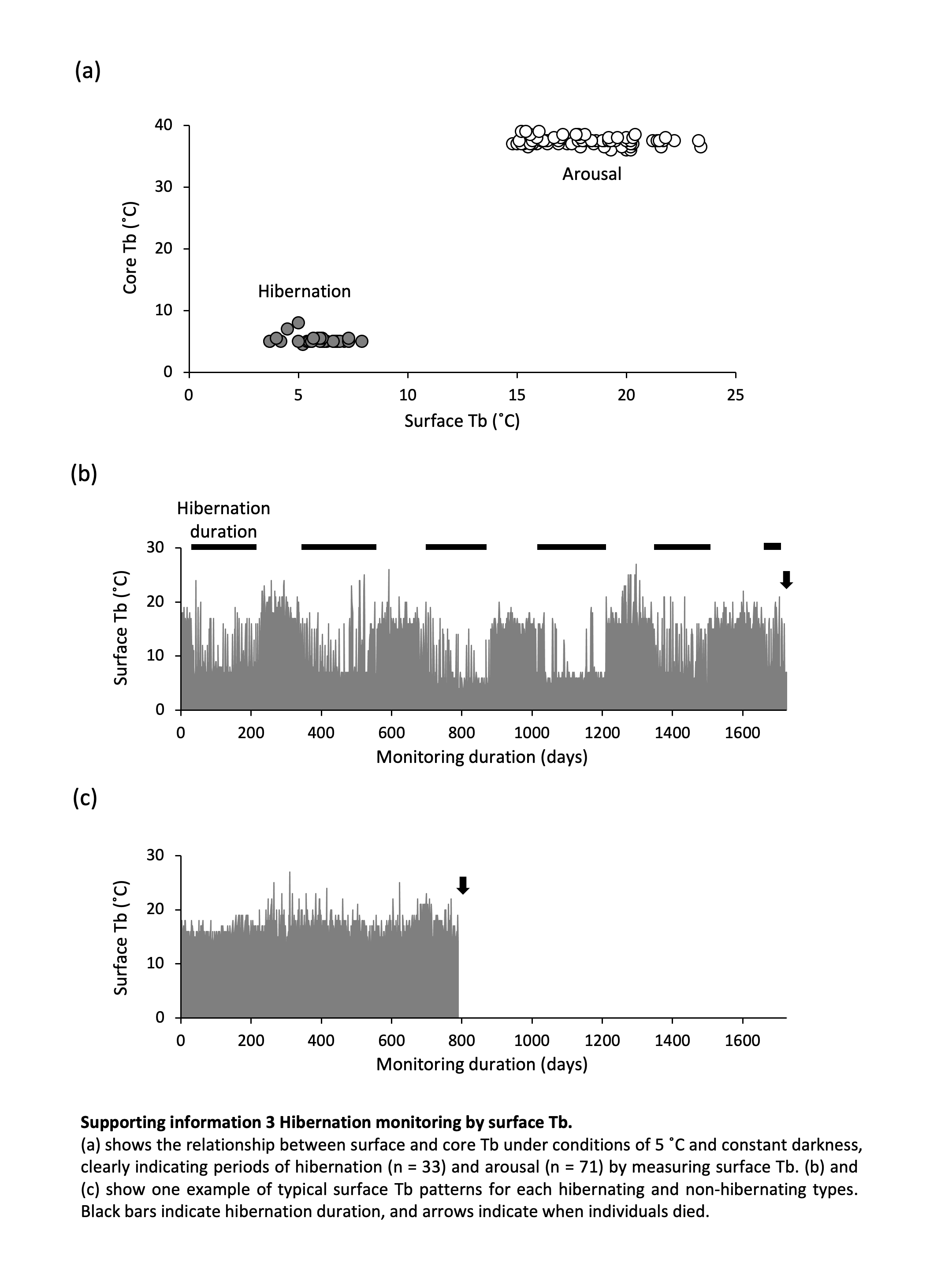

Supplement: Supplementary file 1 [file Image3.TIFF]

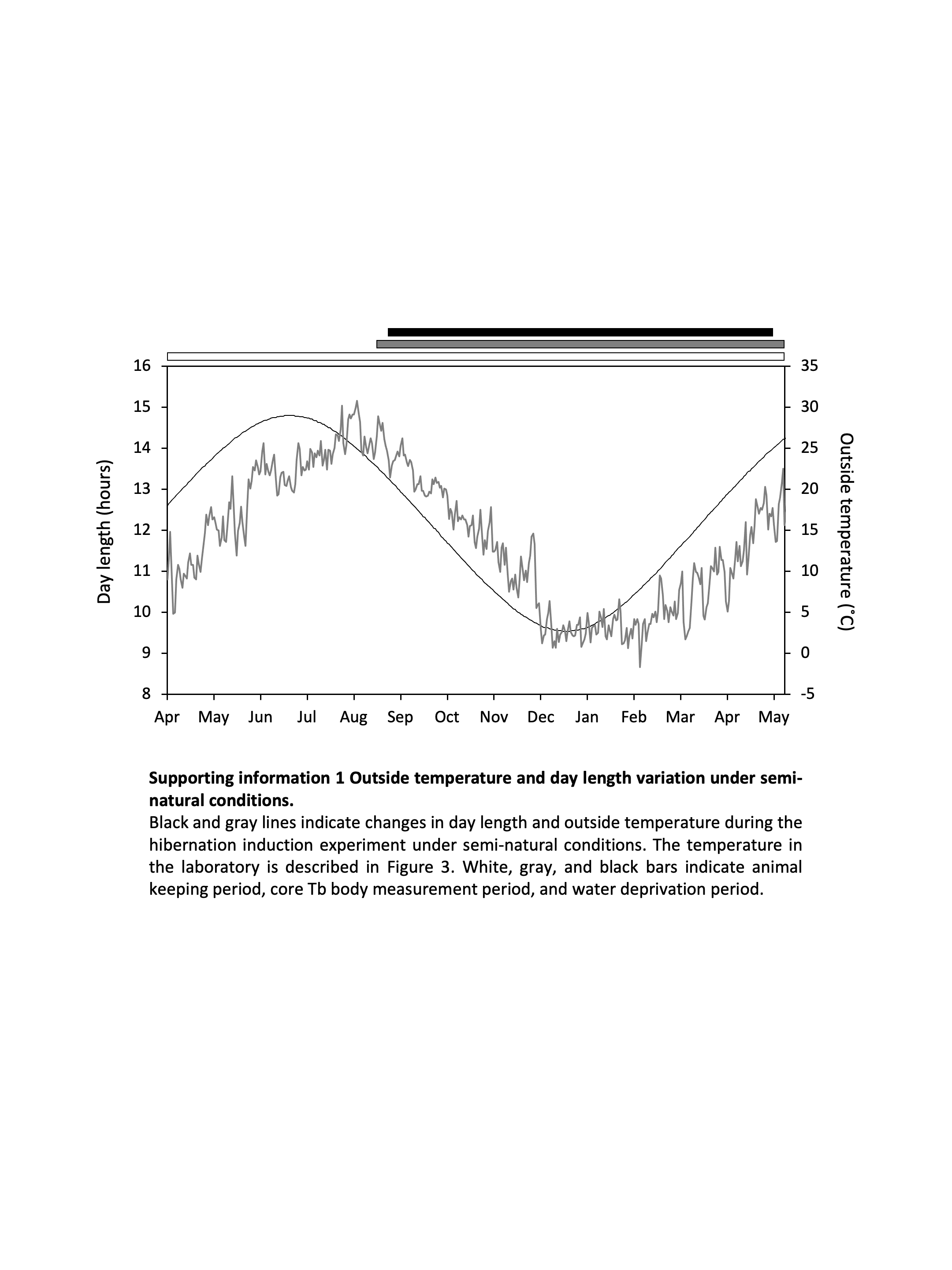

Supplement: Supplementary file 2 [file Image1.TIFF]

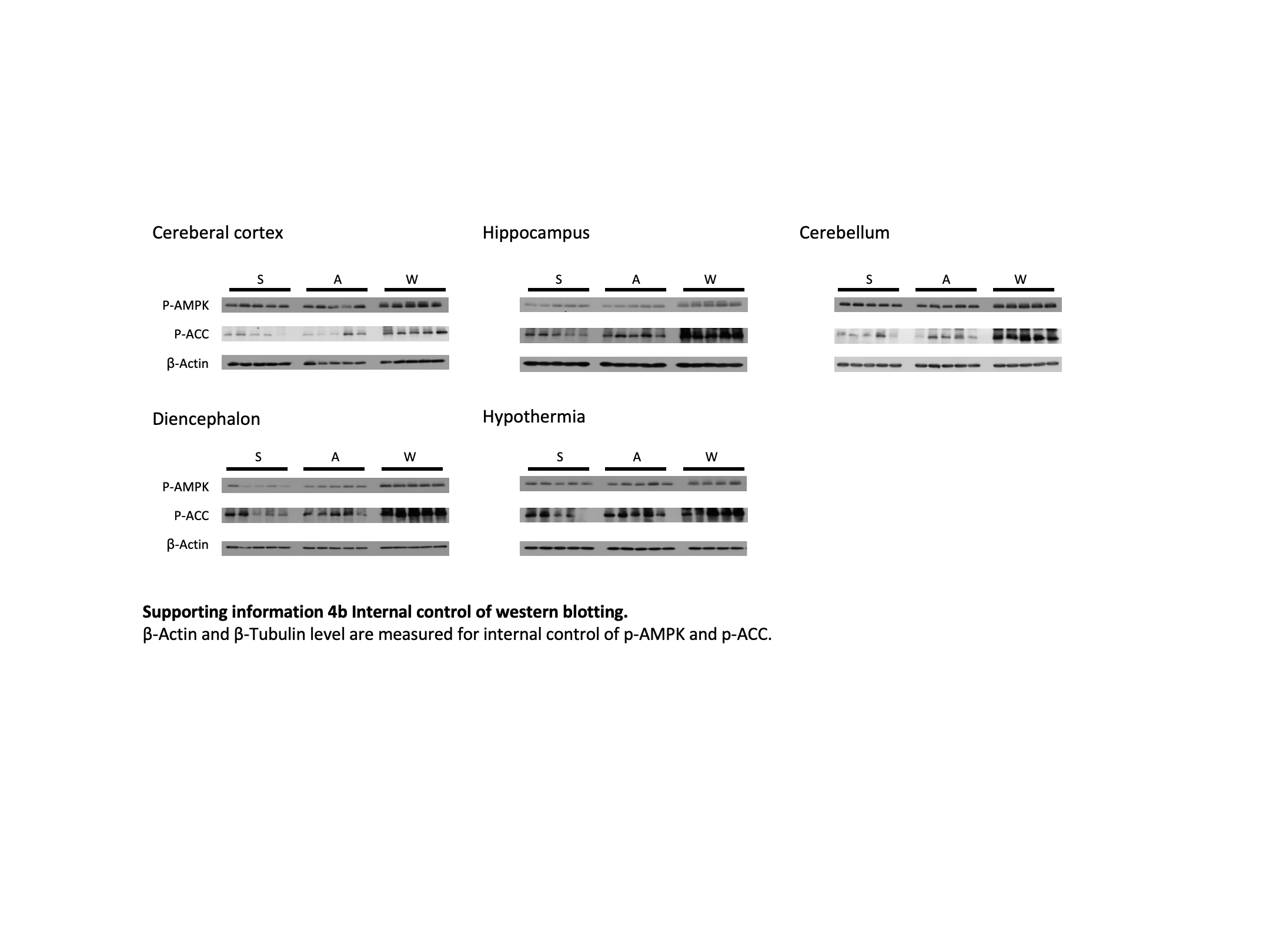

Supplement: Supplementary file 3 [file Image5.TIFF]

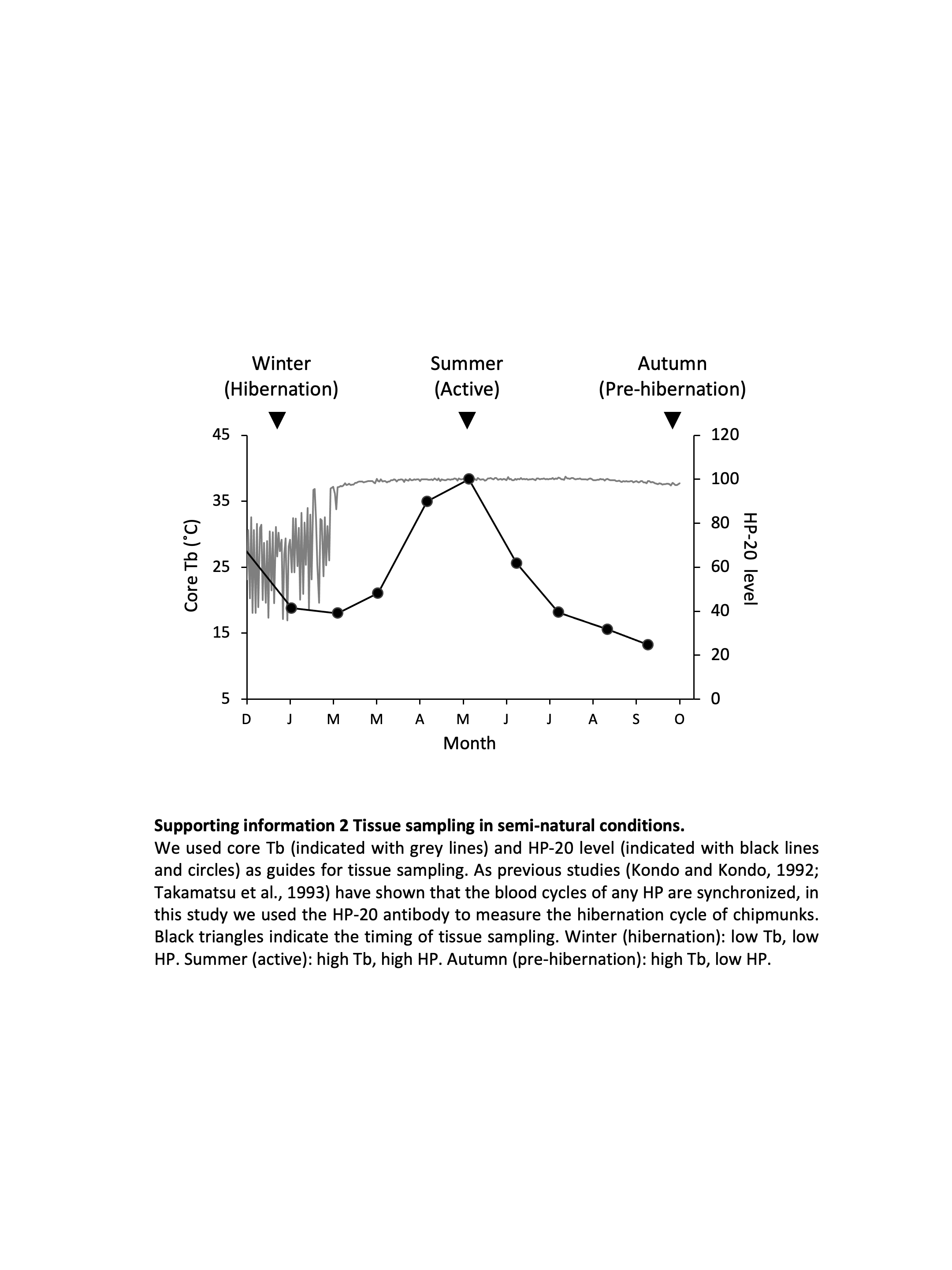

Supplement: Supplementary file 4 [file Image2.TIFF]

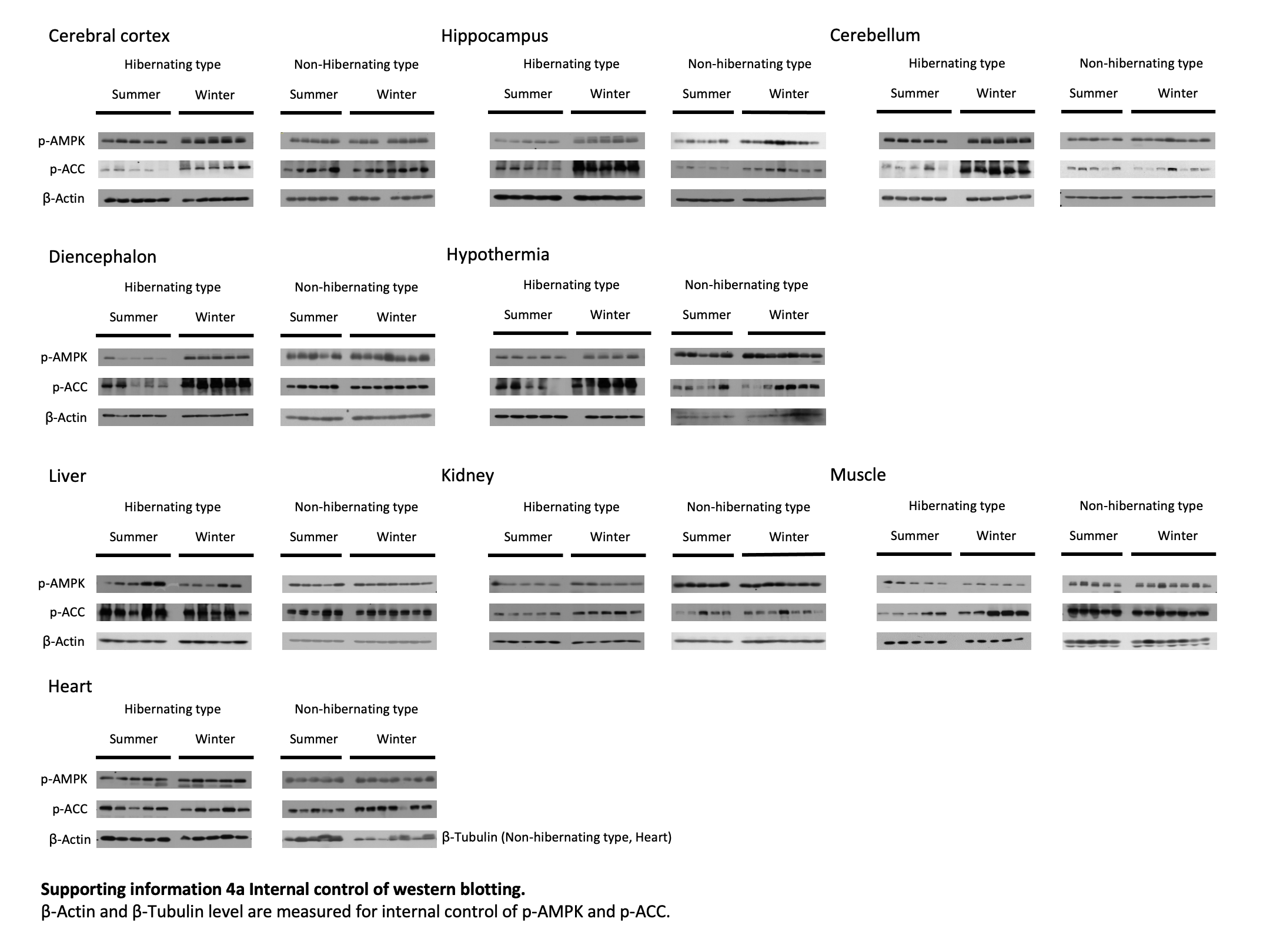

Supplement: Supplementary file 5 [file Image4.TIFF]
